# Social Return on Investment of Home Exercise and Community Referral for People With Early Dementia

**DOI:** 10.1177/23337214221106839

**Published:** 2022-06-03

**Authors:** Ned Hartfiel, John Gladman, Rowan Harwood, Rhiannon Tudor Edwards

**Affiliations:** 1 1506 Bangor University, Bangor, UK; 2 6123 University of Nottingham, Nottingham, UK

**Keywords:** social return on investment, dementia, exercise, physical activity, community referral, social prescribing

## Abstract

Exercise can improve physical function and slow the progression of dementia. However, uncertainty exists around the costeffectiveness of exercise programmes for people with early dementia. The aim of this study was to determine whether a home-based supervised exercise programme (PrAISED – promoting activity, independence, and stability in early dementia) could generate a positive social return on investment (SROI). SROI analysis was conducted as part of a randomised controlled feasibility trial comparing PrAISED with usual care. Wellbeing valuation was used to compare the costs of the programme with the monetised benefits to participants, carers, and healthcare service providers. The PrAISED programme generated SROI ratios ranging from £3.46 to £5.94 for every £1 invested. Social value was created from improved physical activity, increased confidence, more social connection and PrAISED participants using healthcare services less often than usual care. This study found that home-based supervised exercise programmes could generate a positive SROI for people with early dementia. Trial registration: ClinicalTrials.gov: NCT02874300 (first posted 22 August 2016), ISRCTN: 10,550,694 (date assigned 31 August 2016).

## Introduction

Dementia is a common and debilitating disease resulting in high costs to individuals and society. Public Health England has recognised dementia as a major priority. Studies show that delaying the onset of dementia by 2 years can have substantial economic and societal benefits (Rakesh et al., 2017). Research indicates that interventions that promote exercise and physical activity may slow the rate of dementia progression. A study from Finland reported that a twice-weekly, 12-month, supervised exercise programme at home significantly reduced the rate of decline in activities of daily living for people with dementia (Pitkala et al., 2013). This study reported reductions in hospital admissions and overall costs, suggesting that intensive exercise, with the right support, may be achievable and sustainable for improving the quality of life for people with dementia.

PrAISED is a novel intervention in the UK to determine whether an individually tailored, home-based supervised exercise programme for people with early dementia can be clinically effective and cost-effective (Harwood et al., 2018). In alignment with World Health Organisation guidelines, the PrAISED exercise programme involves a minimum of 150 minutes of moderate to vigorous physical activity per week. In addition, the PrAISED programme involves referring people with early dementia to relevant community groups to encourage physical activity and social participation.

Between September 2016 and March 2018, a feasibility randomised controlled trial (RCT) of PrAISED was conducted at two sites, Derby and Nottingham. Patients with early dementia were randomised to a PrAISED programme consisting of moderate to high supervision (12–50 home sessions within 12 months), or to a usual care group consisting of a brief assessment of falls prevention (1 or two sessions within 12 months). Delivered by physiotherapists, occupational therapists and rehabilitation support workers, the PrAISED programme included assessment of patients, creation of individualised exercise plans, delivery of supervised exercises and activities, and referral to appropriate community activities. To encourage good habit-formation and the continuation of self-directed exercise, home visits were tapered over the 12-month study period with more frequent sessions during the first 3 months.

Patients with early dementia were assessed at baseline and 12 months. The primary outcome measure was activities of daily living (ADLs) measured by the Disability Assessment for Dementia (DAD) scale. Secondary outcomes included physical activity, balance, mobility, fear of falling, quality of life, carer strain, and health service use. Additional methodological details are found in the study protocol (Harwood et al., 2018) and the feasibility trial findings relating to effectiveness found elsewhere (Goldberg et al., 2019). A social return on investment (SROI) analysis of the feasibility trial was specified in the study protocol and undertaken to help assess cost-effectiveness and potential value for money.

## Methods

SROI is a pragmatic form of Social Cost–Benefit Analysis (Social CBA), which is recommended in the HM Treasury Green Book to assess interventions that impact social welfare (New Economics Foundation, 2012). Using quantitative and qualitative methods, Social CBA measures and values all relevant costs and outcomes (HM Treasury, 2018). First documented in the early 2000s, SROI methodology has been refined and described in the Cabinet Office ‘A Guide to Social Return on Investment’ (Cabinet Office, 2012). SROI considers what outcomes are relevant and significant to stakeholders and then assigns financial proxies to outcomes which often do not have market values enabling findings to be reported in a common metric (£s).

Using a benefit-cost ratio comparing the value of outcomes with the value of inputs, the reporting of findings is easy to interpret and understandable to a wide audience. With the introduction of the Public Services (Social Value) Act 2012, the UK government is increasingly interested in evaluation methods which capture the social, economic and environmental outcomes of health and social care interventions. In taking a societal perspective, SROI considers the costs and benefits to key stakeholders. A societal approach is useful for informing decision-making within the NHS, which has budgets to be allocated between various programmes.

In this paper, SROI methodology was used to generate a range of SROI ratios using quantitative and qualitative data collected during the PrAISED feasibility trial. SROI methodology involves the following stages: identifying stakeholders, developing a theory of change, evidencing outcomes, valuing outcomes, calculating costs and estimating the SROI ratio. The SROI analysis in this study was conducted in accordance with the 21-item SROI Quality Assessment Framework Tool (Appendix 1).

The first stage was to identify the key stakeholders, which are the people or organisations significantly affected by the PrAISED programme. The PrAISED feasibility study research team, which included patient and public involvement representatives, identified three key stakeholders: patient participants, carer participants and the NHS. It was expected that patient participants would benefit from PrAISED exercises and community referrals; carer participants would benefit from the additional support provided by home visits from PTs, OTs and RSWs; and the NHS would benefit from less frequent health service use from patients participating in the PrAISED programme.

**Figure 1.**
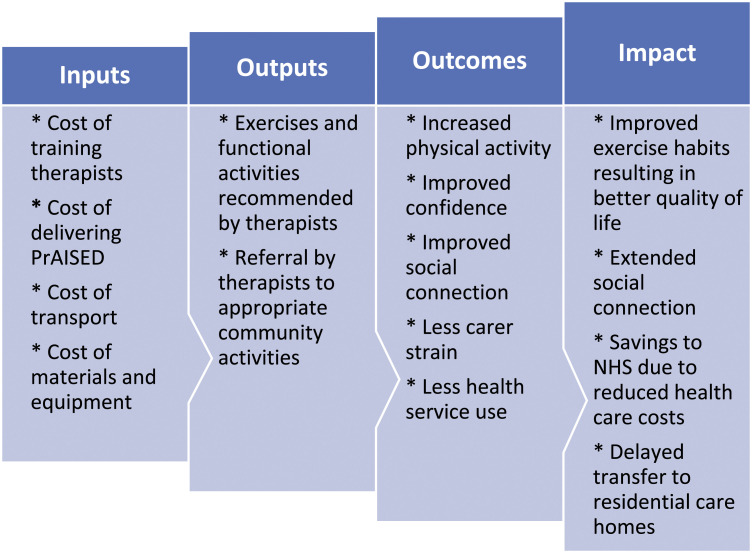
Theory of change model.

A Theory of Change (ToC) model was created to identify the expected benefits experienced by key stakeholders. The ToC model illustrates the links between the inputs, outputs, outcomes and impacts of PrAISED (Figure 1).

After written informed consent was obtained from all participants, qualitative and quantitative data was collected to determine the effectiveness of PrAISED. Interviews were held with patients and carers after the 12-month study. In addition, patients and carers completed baseline and 12-month questionnaires, which measured activities of daily living (ADL), physical activity, balance, mobility, fear of falling, quality of life, carer strain, and health care service use. Evidencing outcomes involved determining the amount of benefit experienced by the three key stakeholder groups: patient participants, carer participants, and the NHS (Table 1)

**Table 1.** Outcome Measures for Three Key Stakeholders.

Stakeholder	Outcome Measure	Completed by	Outcome
Patient participant	Disability assessment for dementia scale (DAD)	Carer participant	ADLs
Patient participant	International physical activity questionnaire	Carer participant	Physical activity
Patient participant	Berg balance scale	Patient participant	Balance
Patient participant	Timed up and go (TUG) test	Patient participant	Balance and mobility
Patient participant	Falls efficacy scale – International (FES-I)	Carer participant	Fear of falling
Patient participant	Health-related quality of life (EQ5D-3L)	Patient participant	Health-related quality of life
Carer participant	Carer strain index (CSI)	Carer participant	Carer strain
NHS	CSRI form	Carer participant	Costs of health care resource use

For patient participants and carer participants, two different outcome scenarios were estimated: a base case and a conservative case. The base case considered patient or carer participants who stayed the same or improved over the 12-month study period for a particular outcome. Because dementia is a progressive condition, no change in outcome over 12 months can be considered a positive result. The conservative case, however, counted only those patient or carer participants who improved for a particular outcome over 12 months.

The next stage of SROI methodology was to determine the value of outcomes for each of the three key stakeholder groups. To monetise the outcomes for patient and carer participants, financial proxies were assigned from the Social Value Bank (SVB), which is a databank of methodologically consistent unit costs for outcome indicators (Trotter et al., 2014). SVB is based on ‘wellbeing valuation’, which is recognised in HM Treasury’s Green Book as a robust method for financial appraisal and evaluation.

Wellbeing valuation uses thousands of large UK national surveys to isolate specific variables and to determine the effect of those variables on a person’s wellbeing. Wellbeing valuation then establishes the equivalent amount of money needed to increase a person’s wellbeing by the same amount. In this study, wellbeing valuation was applied to quantify and monetise significant patient and carer participant outcomes for both the base case and conservative case scenarios.

Total costs of the PrAISED programme included the following categories: training costs for therapists; transportation and staffing costs required for therapists to deliver the programme in patient homes, and equipment costs for instructional materials and exercise equipment.

SROI ratios for base case and conservative case scenarios were generated by dividing the social value per participant by the cost per participant. To avoid overestimating the SROI ratio, ‘deadweight’ (i.e., quantity of outcomes that would have happened anyway) is usually considered (Cabinet Office, 2012). However, due to the randomised controlled study design used in the feasibility trial, calculating deadweight was not necessary as the data collected from the usual care patient participants represented ‘what would have happened anyway’. Discounting was also not necessary in this evaluation as all benefits measured were within 1 year of the baseline assessment.

## Results

After receiving NHS ethics approval, participant recruitment occurred between September 2016 and March 2017 (Goldberg et al., 2019). Early dementia patients (*n* = 60) and carers (*n* = 54) were enlisted from memory assessment clinics, community health venues and online registers. The mean Standardised Mini-Mental State Examination (SMMSE) score for patient participants was 25.6/30. The mean age of participants was 76 years for patients and 68 years for carers. Patient participants were mostly male (57%) and carer participants were mostly female (65%).

Between September 2017 and March 2018, 19 PrAISED patients (with their carers) completed interviews (Appendix 1). Of the 60 patient participants, 82% (49/60) completed both baseline and 12-month questionnaires, which made it possible to determine the percentage who improved, stayed the same, or worsened for each outcome measure. A significant outcome was based on a >10% difference between the percentage of PrAISED and usual care participants who improved for each outcome at 12-months. At 12 months, four significant outcomes were identified:

### Outcome 1: Increased Physical Activity Resulting in Improved Activities of Daily Living

At 12-months, the base case indicated that 43% (12/28) of PrAISED patient participants reported no deterioration or improved DAD scores compared with 21% (3/14) of usual care participants. The conservative case showed 25% (7/28) of PrAISED patient participants with improved DAD scores compared to 14% (2/14) of usual care participants (Table 2).

**Table 2.** Valuing Outcomes for Patient and Carer Participants.

Outcome Scenario	Participant Group	Outcome	Indicators	Quantity	Financial Value from Social Value Bank	Social Value per year	Social Value per Person
Base case	PrAISED	Physical activity	DAD	12/28 (43%) no deterioration or improved	£3537 per year - frequent mild exercise	£42,444	£1516
Conservative case	PrAISED	Physical activity	DAD	7/28 (25%) improved	£3537 per year - frequent mild exercise	£24,759	£884
Base case	Usual care	Physical activity	DAD	3/14 (21%) no deterioration or improved	£3537 per year - frequent mild exercise	£10,611	£758
Conservative case	Usual care	Physical activity	DAD	2/14 (14%) improved	£3537 per year - frequent mild exercise	£7074	£505
Base case	PrAISED	Improved confidence	FES-I	15/26 (58%) no deterioration or improved	£13,080 per year - feeling high confidence	£196,200	£7546
Conservative case	PrAISED	Improved confidence	FES-I	8/26 (31%) improved	£13,080 per year - feeling high confidence	£104,640	£4025
Base case	Usual care	Improved confidence	FES-I	4/12 (33%) no deterioration or improved	£13,080 per year - feeling high confidence	£52,320	£4360
Conservative case	Usual care	Improved confidence	FES-I	3/12 (25%) improved	£13,080 per year - feeling high confidence	£39,240	£3270
Base case	PrAISED	Social connection	EQ5D-3L	22/29 (76%) no deterioration or improved	£3753 per year - sense of belonging	£82,566	£2847
Conservative case	PrAISED	Social connection	EQ5D-3L	15/29 (52%) improved	£3753 per year - sense of belonging	£56,295	£1941
Base case	Usual care	Social connection	EQ5D-3L	6/13 (46%) no deterioration or improved	£3753 per year - sense of belonging	£22,518	£1732
Conservative case	Usual care	Social connection	EQ5D-3L	2/13 (15%) improved	£3753 per year - sense of belonging	£7506	£577
Base case	PrAISED	Less carer strain	CSI	17/25 (68%) no deterioration or less strain	£6784 per year – able to rely on family	£115,328	£4613
Conservative case	PrAISED	Less carer strain	CSI	10/25 (40%) less strain	£6784 per year – able to rely on family	£67,840	£2714
Base case	Usual care	Less carer strain	CSI	5/12 (42%) no deterioration or less strain	£6784 per year – able to rely on family	£33,920	£2827
Conservative case	Usual care	Less carer strain	CSI	3/12 (25%) less strain	£6784 per year – able to rely on family	£20,352	£1696
Outcome 1: Physical activity - difference between groups (base case)							£758
Outcome 1: Physical activity - difference between groups (conservative case)							£379
Outcome 2: Confidence - difference between groups (base case)							£3186
Outcome 2: Confidence - difference between groups (conservative case)							£755
Outcome 3: Social connection - difference between groups (base case)							£1115
Outcome 3: Social connection- difference between groups (conservative case)							£1364
Outcome 4: Carer strain - difference between groups (base case)							£1786
Outcome 4: Carer strain - difference between groups (conservative case)							£1018

### Outcome 2: More Confidence Resulting in Less Fear of Falling

At 12-months, 58% (15/26) of PrAISED patient participants reported no change or less concern about falling compared with 33% (4/12) of usual care patient participants. The conservative case indicated 31% (8/26) of PrAISED patient participants with improved falls efficacy scores compared to 25% (3/12) of usual care participants (Table 2).

### Outcome 3: More Social Connection Resulting in Improved Quality of Life

At 12-months, 76% (22/29) PrAISED patient participants reported no change or improved health-related quality of life compared with 46% (6/13) of usual care patient participants. The conservative case indicated 52% (15/29) of PrAISED patient participants with improved EQ5D scores at 12-months compared to 15% (2/13) of usual care patient participants (Table 2).

### Outcome 4: Less Carer Strain (for carer participants)

At 12-months, 68% of PrAISED carer participants reported no deterioration or less carer strain compared to 42% of usual care participants. The conservative case indicated 40% (10/25) of PrAISED carers with reduced carer strain compared to 25% (3/12) of usual care carers (Table 2).

Outcomes for the NHS were assessed by measuring the health service use of patient participants during the 12-month study. Health service use for patient participants was completed by carer participants at baseline and 12 months via a Client Service Receipt Inventory (CSRI) form. Health service use included contact with GPs, nurses, outpatient services, accident and emergency (A&E) services, inpatient hospital days, physiotherapists and occupational therapy services. The 12-month results indicated that PrAISED patient participants used NHS health services less than the usual care patient participants by an average of £1179 per person during the study (Table 3).

**Table 3.** Valuing Outcomes for the NHS.

Health Service Use	Baseline	12-mos	Quantity Difference	Cost per Visit	Total Cost	Average Cost per Patient
PrAISED Patients (*n* = 33)						
Usual Care Patients (*n* = 16)						
GP visits (PrAISED)	48	38	−10	£37/visit^a^	-£370	-£11.21
GP visits (usual care)	18	23	+5	£37/visit^a^	+£185	+11.56
Nurse visits (PrAISED)	51	50	−1	£36/visit^a^	-£36	-£1.09
Nurse visits (usual care)	14	8	−6	£36/visit^a^	-£216	-£13.50
Outpatient services (PrAISED)	51	23	−28	£125/visit^b^	-£3500	-£106.06
Outpatient services (usual care)	28	12	−16	£125/visit^b^	-£2000	-£125.00
A&E services (PrAISED)	4	5	+1	£160/visit^b^	+£160	+£4.85
A&E services (usual care)	0	2	+2	£160/visit^b^	+£320	+£20.00
Inpatient hospital days (PrAISED)	1	15	+14	£1603/day^b^	£22,442	+£680.06
Inpatient hospital days (usual care)	0	18	+18	£1603/day^b^	£28,854	+£1803.38
PT/OT (PrAISED)	6	4	−2	£44/visit^a^	-£88	-£2.67
PT/OT (usual care)	4	21	+17	£44/visit^a^	+£748	+46.75
Total (PrAISED)						£564
Total (usual care)						£1743
Difference between groups						£1179

^a^ Curtis & Burns, 2018

^b^ NHS Improvement, 2018

The NHS was responsible for the training and delivery costs for the 12-month PrAISED programme. The equivalent of one full-time therapist (PT/OT) and four full-time RSWs delivered the PrAISED programme to 33 patient participants over the 12-month study period. The therapist and RSWs received two full days of PrAISED training prior to the start of the feasibility study. Training costs were estimated at £583 per therapist/RSW (Appendix 2). The difference in training and delivery costs between PrAISED and usual care patient participants was estimated at £1351 (Appendix 2).

The SROI ratio for the base case was £5.94: £1, meaning that £5.94 of social value was generated for every £1 invested in the programme (Table 4). When the conservative case was considered, the total social value per participant was £4672 compared with £8024 in the base case, and the social value ratio was £3.46 of social value generated for every £1 invested in the programme (Table 4).

**Table 4.** Social Return On Investment Ratios.

Cost Scenario	Category	Difference Between Groups
Base case	Outcome 1 – increased physical activity	£758 per participant
Conservative case	Outcome 1 – increased physical activity	£379 per participant
Base case	Outcome 2 – improved confidence	£3186 per participant
Conservative case	Outcome 2 – improved confidence	£755 per participant
Base case	Outcome 3 – additional social connection	£1115 per participant
Conservative case	Outcome 3 – additional social connection	£1341 per participant
Base case	Outcome 4 – less carer strain	£1786 per participant
Conservative case	Outcome 4 – less carer strain	£1018 per participant
	Outcomes for NHS	£1179 per participant
Base case	Total social value for all stakeholders	£8024 per participant
Conservative case	Total social value for all stakeholders	£4672 per participant
Base case	Total cost	£1351 per participant
Conservative case	Total cost	£1351 per participant
Base case	SROI benefit/cost ratio	£5.94: £1
Conservative case	SROI benefit/cost ratio	£3.46: £1

## Discussion

The results indicated that the PrAISED programme generated a positive SROI ratio ranging from £3.46 (conservative case) to £5.94 (base case) for every £1 invested. These ratios (£3.46 - £5.94: £1) were similar to SROI ratios generated in other related studies. For example, a study of peer support groups for people with dementia generated social value ratios ranging from £1.17 to £5.18 for every £1 invested (Willis et al., 2018); an evaluation of arts activities for people with dementia revealed social value ratios ranging from £3.20 to £6.62 for every £1 invested (Jones, Windle, et al., 2020); and a physical activity intervention for older people with chronic health conditions showed SROI ratios ranging from £2.60 to £5.16 for every £1 invested (Jones, Hartfiel, et al., 2020).

In this SROI of PrAISED, patient participants gained social value from improved physical activity, higher confidence, and more social connection. Carer participants acquired social value from less carer strain. The NHS obtained social value from reduced demand on health services by patient participants during the 12-month study.

In the base case scenario, greater percentages of social value were derived from patient participants experiencing higher confidence (40% of total social value), carer participants reporting less carer strain (22% of total social value), and the NHS benefitting from reduced demand on health services (15% of total social value).

In the conservative case, greater percentages of social value were derived from patient participants experiencing more social connection (29% of total social value), the NHS benefitting from reduced demand on health services (25% of total social value) and carer participants reporting less carer strain (22% of total social value).

Although the PrAISED intervention focused on providing supervised physical activity at home, greater percentages of social value were due to mental and social outcomes (i.e., higher confidence, less carer strain, and more social connection) experienced by patient participants and their carer. Financial values in the Social Value Bank indicate that mental health has a high impact on overall wellbeing. Home visits by PrAISED therapists and subsequent referral to community activities enabled patient participants to keep socially connected to the wider community, which can be crucial for sustaining mental wellbeing and quality of life (Birt et al., 2020).

### Strengths of This Study

Although previous SROI evaluations have investigated the social value of arts activities and peer support groups for people with dementia, this is the first study to estimate the social value of physical activity and community referral to people with early dementia and their carer. A strength of this evaluation is the societal perspective and the inclusion of multiple stakeholder groups. Second, the results were strengthened by the randomised controlled study design, which is rare in SROI evaluations. Third, the validity of the results was strengthened from both quantitative and qualitative data collected from baseline and follow-up questionnaires completed by patients and their carer. Finally, the social value ratios calculated in this study were generated from value sets derived from wellbeing valuation, a consistent and robust method recommended in HM Treasury’s Green Book (2018) for measuring social CBA.

### Limitations of This Study

First, there are only a limited number of pre-determined outcome values in the SVB derived from wellbeing valuation (Social Value UK, 2015). Not all outcome values in the SVB fit perfectly with the outcomes measured in health-related interventions. For example, high confidence in the SVB is valued at £13,080 per person per year. In our study, high confidence was derived from improvement in the Falls Efficacy Scale and applied to those patient participants who became more confident in walking and doing daily activities. SVB values, however, are typically binary, meaning that they are either applied in full or not at all. SVB valuations do not allow for situations where there may be varying degrees of improvement (Social Value UK, 2015).

Second, a common issue is that social value researchers working with the same data may arrive at different SROI ratios (Fujiwara, 2015). More social connection, for example, could be matched in the SVB with either ‘feeling belonging to neighbourhood’ (£3753 per person per year) or ‘member of a social group’ (£1850 per person per year). Matching outcomes from the study data with the most appropriate SVB value depends on the researcher’s discretion. This can introduce potential researcher bias and the possibility that social value estimates are upward-biased (Fujiwara, 2015).

Third, the PrAISED programme was individualised. Although many of the exercises prescribed were the same, each patient received a personalised programme involving home exercises and referral to relevant community activities. Due to the specific skills of therapists and RSWs, and to the varied needs and abilities of patients, the individual PrAISED programmes differed considerably in the content delivered. For example, participant benefit could have been due to the PrAISED exercises or from community activities such as the parkrun, dementia-friendly swimming or ballroom dancing. The variety of activities prescribed makes it inappropriate to determine whether particular PrAISED activities or referrals were most responsible for participant outcomes.

Finally, although the SROI analysis was mentioned in the feasibility trial protocol (Harwood et al., 2018), the methods of analysis were not pre-specified. In addition, the SROI ratios reported in this study need validation from larger multi-centre trials with higher sample sizes.

## Conclusion

This SROI analysis is based on quantitative and qualitative data from the PrAISED feasibility study with 60 patient participants and 54 carer participants. The results showed that a home-based supervised exercise programme with ongoing referral to community activities could generate a positive SROI ratio for people with early dementia. By delivering positive social value for money, PrAISED can help people with early dementia stay more independent, active, and socially connected. To confirm these results, a further SROI analysis will be required on the larger multi-centred randomised controlled trial of the PrAISED programme currently in progress (Bajwa et al., 2019). In addition, future SROI analysis could compare the social value ratios generated from face-to-face and online delivery of PrAISED.

## Appendix 1 Selected Quotes from Interviews with Patients and Carers Indicating Physical, Mental and Social Benefits of Participation in PrAISED

### Physical Benefits

• ‘The physio exercises are important, because it’s keeping me fit’ (Patient participant, 1001).
• ‘My joints get a bit stiff these days…the exercises help to ease them.’ (Patient participant 1006)
• ‘With the help of the swimming (Alzheimer’s Group) and the other exercises, that’s given me more balance and strength.’ (Patient participant 1009)
• ‘Instead of doing the exercises, we’d go out for a walk. But the physio did suggest that, whilst you go out for your walks, you’re sort of implementing some of the exercises.’ (Patient participant 1011).
• ‘But that was the breakthrough, she took me out, she ran me up and down and said, you can run, do the park run. And that was it. And I’ve done 39 now.’ (Patient participant 1017).
• ‘Yes, the programme has encouraged me to walk more and exercise more. I feel better for it. I walk to the shop and back again, trying to get the steps in.’ (Patient participant 1018)
• ‘You keep doing it and it works. It gets you back doing exercise. I used to play a lot of sport and got out of the habit, but it kept me going again.’ (Patient participant 1026)
• ‘It’s proved the benefits of those specific PrAISED exercises. His actual gait, when he walks, is a lot better than it was a year ago.’ (Carer participant for 1026)
• ‘The exercises made my back a lot better than it was.’ (Patient participant 2007)

### Mental Benefits

• ‘I feel great after all the exercises. Feel as though I’ve achieved something.’ It’s the activity from and the encouragement from PrAISED that has given me the confidence to go swimming (Alzheimer’s group). I’ve had positive encouragement from the group and quite a lot of improvements. The programme has given me a positive attitude.’ (Participant 1009)
• ‘It’s given me a boost, for a start, a very big boost.’ (Patient participant 1017)
• ‘I do different exercises. I go walking, I do the exercises and I do the gardening, and they’re all part and parcel. I was doing things that I never thought I could do.’ (Patient participant 1018).
• ‘It got you doing that life journey scrapbook, that’s something you wouldn’t have done. So then, Alzheimer’s had a carol concert, and they said would you give a talk at that? So you stood up with a mic in front of you, and you talked on living well with dementia.’ (Carer for patient participant 2012)

### Social Benefits

• ‘The people (therapist and RSW) who came were all very helpful and kind.’ (Patient participant 1017)
• ‘The parkrun has a social aspect to it. They’ve got to know me over there and chat to me and, it always pleases me because I come first in my age group.’ (Patient participant 1017)
• ‘SG (therapist or RSW) was here every week. And she loved walking. And she put me through my paces. And that was motivation for me, that I were going to beat her, I think I did a couple of times but not very often. And it’s, it’s embedded in me now, to carry on doing it. And I wish I could get her (therapist or RSW) to come again and do it for another year with me.’ (Patient participant 1018)
• ‘So he’s snooker on a Monday. Tuesday, you have been going to Man Cave. He started dementia-friendly swimming at the leisure centre. Thursday, we go to Healthy Hearts exercise class. Friday, you have been doing table tennis. And Sunday night, we’ve started ballroom dancing. So, that is how our life is changed because he found he could still do things.’ (Carer participant for 2012).

## Appendix 2 Supplementary Tables for Training and Delivery Costs

### Training Costs for Two-Day Course (2 Trainers, 1 Therapist and 4 RSWs)

**Table:**

Cost Categories for Training	Cost
Cost of two trainers (£195.17 per day per trainer x 2 trainers x 2 days)	£781
Cost of mileage for 7 people (2 trainers +5 therapists) (25 miles average roundtrip x £0.45 per mile)	£158
Cost of manuals and printing for 5 therapists over 2-day training (£12.50 per person x 5)	£63
Cost of 1 therapist attending 2-day training (£195.17 per day per therapist x 1 therapists x 2 days)	£390
Cost of 4 RSWs attending 2-day training (£122.82 per day per RSW x 4 RSWs x 2 days)	£983
Cost of lunch and refreshments for 7 people (7 people x 2 days x £10 for lunch and refreshments)	£140
Cost of venue hire (£200 per day x 2 days) = £400	£400
**Total costs to deliver 2-day training for 7 people (2 trainers + 5 therapists)**	**£2915**
**Average cost of a 2-day training per therapist/RSW**	**£583**

### Delivery Costs for 60 Patient Participants

**Table:**

Relevant Information for Calculating Delivery Costs	PrAISED	Usual Care
Number of participants with early dementia receiving PrAISED intervention	33	16
Average number of sessions delivered per participant	23	2
Total number of sessions delivered	759	32
Average time for therapist/RSW to deliver a session (including travel)	2.5 hours	2.5 hours
Total number of hours delivering PrAISED	1898 hours	80 hours
Average number of sessions delivered per participant by registered therapists	5 (22%)	1 session
Average number of sessions delivered per participant by support workers	18 (78%)	1 session
Total number of registered therapist hours	418 hours	40 hours
Total number of support worker hours	1480 hours	40 hours
Average mileage to and from participants’ homes (round trip)	25 miles	25 miles
Total mileage to and from participants’ homes	18,975 miles	800 miles
Total cost of mileage to and from participants’ homes (£0.45 per mile)	£8539	£360
Total cost of registered therapists (£26.92/hour)	£11,253	£1077
Total cost of support workers (£16.94/hour)	£25,071	£678
Equipment costs (£30 per participant)	£990	£0
Stationery costs (£5 per participant)	£165	£0
Training costs for one therapist and four RSWs	£2915	£0
TOTAL COSTS	£48,933	£2115
TOTAL COSTS PER PARTICIPANT	£1483	£132
**DIFFERENCE IN TOTAL COSTS PER PARTICIPANT BETWEEN GROUPS**	**£1351**	
